# Relationships between Levels of Serum IgE, Cell-Bound IgE, and IgE-Receptors on Peripheral Blood Cells in a Pediatric Population

**DOI:** 10.1371/journal.pone.0012204

**Published:** 2010-08-16

**Authors:** Eleonora Dehlink, Alexandra H. Baker, Elizabeth Yen, Samuel Nurko, Edda Fiebiger

**Affiliations:** 1 Division of Gastroenterology and Nutrition and EGID Centre, Children's Hospital Boston, Harvard Medical School, Boston, Massachusetts, United States of America; 2 Department of Pediatrics and Adolescent Medicine, Medical University of Vienna, Waehringer Guertel, Vienna, Austria; Columbia University, United States of America

## Abstract

**Background:**

Elevated serum immunoglobulin (Ig) E is a diagnostic marker of immediate-type allergic reactions. We hypothesize that serum IgE does not necessarily reflect total body IgE because *in vivo* IgE can be bound to cell surface receptors such as FcεRI and FcεRII (CD23). The aim of this study was to analyze the relationships between levels of serum IgE, cell-bound IgE, and IgE-receptors on peripheral blood cells in a pediatric population.

**Methodology:**

Whole blood samples from 48 children (26 boys, 22 girls, mean age 10,3±5,4 years) were analyzed by flow cytometry for FcεRI, CD23, and cell-bound IgE on dendritic cells (CD11c+MHC class II+), monocytes (CD14+), basophils (CD123+MHC class II-) and neutrophils (myeloperoxidase+). Total serum IgE was measured by ELISA and converted into z-units to account for age-dependent normal ranges. Correlations were calculated using Spearman rank correlation test.

**Principal Findings:**

Dendritic cells, monocytes, basophils, and neutrophils expressed the high affinity IgE-receptor FcεRI. Dendritic cells and monocytes also expressed the low affinity receptor CD23. The majority of IgE-receptor positive cells carried IgE on their surface. Expression of both IgE receptors was tightly correlated with cell-bound IgE. In general, cell-bound IgE on FcεRI+ cells correlated well with serum IgE. However, some patients carried high amounts of cell-bound IgE despite low total serum IgE levels.

**Conclusion/Significance:**

In pediatric patients, levels of age-adjusted serum IgE, cell-bound IgE, and FcεRI correlate. Even in the absence of elevated levels of serum IgE, cell-bound IgE can be detected on peripheral blood cells in a subgroup of patients.

## Introduction

Immunoglobulin (Ig) E and its cell surface receptors, the high affinity receptor FcεRI and the low affinity receptor FcεRII (CD23), are key components of immediate-type allergic reactions [1]. IgE activates the allergic cascade effectively via the high affinity receptor, FcεRI, on blood- and tissue cells. As a member of the immunoglobulin receptor superfamily, FcεRI consists of a ligand-binding immunoglobulin domain-containing protein (α-chain) which binds the Fc-part of IgE and signaling subunits that regulate cellular activation (β- and γ-chains) [2]. Humans express a tetrameric FcεRI (FcεRIαβγ_2_) on mast cells and basophils and a trimeric form (FcεRIαγ_2_) on antigen presenting cells [2], [3]. FcεRI expression is regulated by IgE since binding of monomeric IgE to FcεRIα stabilizes the receptor at the cell surface as an IgE-FcεRI complex [4], [5], [6]. Hence, *in vivo*, individuals with high serum IgE levels show enhanced FcεRI surface expression on peripheral blood basophils, monocytes, and dendritic cells [7], [8], [9].

In the blood, the low affinity IgE receptor CD23 is expressed on B-cells [1], monocytes [10], and dendritic cells [11], [12]. CD23 is a member of the C-type lectin superfamily. Its IgE binding sites consist of 3 lectin-type head domains on triple α-helical coiled-coil stalk domains [1]. The stalk region of CD23 is sensitive to proteolysis, which results in the release of soluble fragments [13], [14]. No correlation between serum IgE and cell surface CD23 levels has been described [10]. In fact, CD23 is being discussed as a regulator of IgE synthesis through a complex interplay of co-ligations of CD23, soluble CD23, IgE, IgE-allergen complexes, and the complement receptor CD21 on B-cells [1].

Serum IgE is an essential laboratory parameter in the diagnosis of allergies. Serum IgE levels generally correlate with the severity of allergic diseases [15], [16], [17]. However, a subgroup of allergic patients has serum IgE levels in the normal range [18], [19]. We therefore hypothesized that serum IgE does not necessarily reflect total body IgE because *in vivo* IgE can be bound to its cell surface receptors FcεRI and CD23. The aim of this study was to analyze the relationships between levels of serum IgE, cell-bound IgE, and IgE-receptors on peripheral blood cells of pediatric patients.

## Materials and Methods

### Study population

This analysis was performed as a substudy within an ongoing prospective cohort study on the role of FcεRI in the gastrointestinal tract. Patients scheduled for an elective esophago-gastro-duodenoscopy at the Division of Gastroenterology at Children's Hospital Boston were randomly invited to participate. Subjects between 1 and 18 years of age were enrolled. Patients' characteristics such as age, gender, and race were obtained in a structured interview. Subjects with a recent (<3 months) use of steroid in any form, immunomodulatory drugs, mast cell stabilizer, or leukotriene inhibitor, as well as patients with an established diagnosis of autoimmune, inflammatory, or immunodeficiency disease and patients under 1 year of age were excluded from the study. The study protocol was approved by the Investigational Review Board at Children's Hospital Boston (Harvard Medical School, Boston, MA). Patients or their legal guardians provided written informed consent. We obtained peripheral blood samples for serum IgE measurements and flow cytometry analysis at the time of enrollment.

### Total serum IgE

Total serum IgE was determined using a solid-phase ELISA (DiaMed Eurogen, Turnhout, Belgium) according to the manufacturer's instructions. In brief, plates pre-coated with an anti-IgE monoclonal antibody were incubated with patient sera and a second anti-IgE antibody conjugated to horseradish peroxidase. After washing, levels of bound IgE were determined by incubating with tetramethylbenzidine (TMB) solution and stopping the reaction with 2N H_2_SO_4_ before reading ODs at 450 nm. Normal serum IgE levels are given by the manufacturer as <10 IU/ml for age 0–3 years, <25 IU/ml for 3–4 years, <50 IU/ml for 4–7 years, <100 IU/ml for 7–14 years, and <150 IU/mL for adults older than age 14. IgE levels above the age specific normal range are referred to as ‘elevated’ IgE from here on.

### Flow cytometry analysis of IgE-receptors and IgE on peripheral blood cells

100 microl of K2EDTA blood were stained following the Becton Dickinson protocol for direct immunofluorescence staining of whole blood (http://www.bdbiosciences.ca/canada/downloads/is_protocols/IF_WB_LW.pdf). The following antibodies were used: anti-IgE FITC (Invitrogen), anti-FcεRIα APC (mAb CRA1, eBioscience, San Diego, CA), anti-CD23 APC, anti-myeloperoxidase (MPO)-PE for neutrophils, anti-CD14 PerCP-Cy5.5 for monocytes, anti-CD11c V450 and anti-MHC class II-PerCP (all BD Biosciences) for dendritic cells. Basophils were identified by anti-CD123-PE (BD) and negativity for MHC class II.

Blood was incubated with antibodies for 20 minutes at room temperature to stain for cell surface IgE, IgE receptors and linage-specific surface markers. Red blood cells were lysed with 1× BD FACS™ Lysing Solution (BD Biosciences, San Jose, CA). After surface staining, neutrophils were identified by intracellular expression of MPO using FIX&PERM® (Invitrogen, Carlsbad, CA) according to the manufacturer's guidelines. Cells were washed and resuspended in PBS for flow cytometry analysis. Corresponding isotype controls were performed for each staining.

Data were acquired with a DakoCytomation MoFlo Legacy (Dako North America Inc., Capinteria, CA) and analyzed with Summit for Windows Version 4.3 (Dako North America Inc.). Typically, 20,000 events within the granulocytes/monocytes gate in the forward/side scatter plot were acquired. FcεRI and CD23 levels were determined as percent (%) positive cells among cell type after subtraction of the individual isotype control background. Cell-bound IgE levels were recorded as the percentage of cells that were double positive for IgE and FcεR1 or IgE and CD23 after subtraction of background.

### Statistical analysis

Data were analysed with SPSS for Windows (version 16.0, SPSS Inc., Chicago, IL). Chi-square test was used for nominal variables. Continuous variables were compared by Student's T-test or Mann-Whitney-U where called for. Correlations were calculated using Spearman's rank correlation test since datasets were not normally distributed. Spearman's rank correlation coefficients are displayed as ‘rho’. To account for age-dependent normal ranges, total IgE levels were converted into z-units as published by Kjellman et al. [20]. A p-value of <0.05 was considered significant.

## Results

A total of 48 pediatric patients were included in the study between October 3, 2008 and July 17, 2009 (26 boys, 22 girls, mean age 10.3±5.4years). The characteristics of the study population are given in Table 1.

**Table 1:** Study population.

Variable	All	normal IgE	elevated IgE	p-value
Subjects, n (%)	48	34 (70,8)	14 (29,2)	
Age, mean±SD	10.3±5.4	11.6±4.5	7.3±6.3	0.033†
Gender				
Male, n (%)	26 (54.2)	19 (55.8)	7 (50)	
Female, n (%)	22 (45.8)	15 (44.1)	7 (50)	0.71‡
Race (n)				
Caucasian	39	28	11	
Asian	1	1	-	
African American	1	-	1	
Caucasian/Asian	2	1	1	
Caucasian/Native American	2	1	-	
N/A	3	2	1	

† Student's T-test,

‡ Chi-Square test. SD  =  standard deviation, N/A  =  not specified.

### Surface FcεRI on peripheral blood dendritic cells and monocytes correlates with serum IgE levels in children with elevated IgE

**Figure 1:**
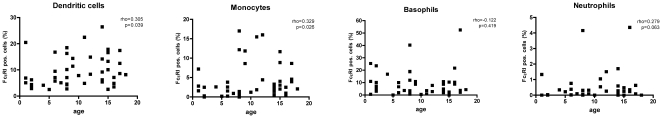
FcεRI expression increases with age in dendritic cells and monocytes. Data points represent percent (%) positive cells among cell type after subtraction of isotype control background in individual children.

All patients expressed FcεRI on their dendritic cells (DCs). FcεRI-expression ranged between 2.54 and 26.43% FcεRI+ DCs (Table 2). The majority of patients also expressed FcεRI on monocytes (44/48), with a range of expression between 0.19 and 17%, basophils (45/48, 0.53–60.17% positive cells), and neutrophils (35/47, 0.1–4.36% positive cells). Among all children, DC and monocyte FcεRI expression slightly increased with age (Figure 1, rho = 0.305, p = 0.039 for DCs, rho = 0.329, p = 0.026 for monocytes).

**Table 2:** Peripheral blood dendritic cells, monocytes, basophils, and neutrophils express FcεRI, CD23 and receptor-bound IgE at the cell surface.

Cell type		N	Median (range) in positive cells
DC (CD11c+)	FcεRI	48/48	8.02 (2.54–26.43)
	CD23	20/48	1.04 (0.03–4.25)
	IgE on FcεRI +	45/48	2.63 (0.02–17.5)
	IgE on CD23+	17/48	0.72 (0.02–4.18)
Monocytes (CD14+)	FcεRI	44/48	2.54 (0.19–17.0)
	CD23	11/48	0.8 (0.29–6.96)
	IgE on FcεRI+	41/48	1.18 (0.1–9.66)
	IgE on CD23+	11/48	0.27 (0.01–3.92)
Basophils (CD123+MHCII-)	FcεRI	45/48	7.22 (0.53–60.17)
	CD23	1/48	0.81
	IgE on FcεRI+	41/48	3.54 (0.01–61.34)
	IgE on CD23+	1/48	1.8
Neutrophils (MPO+)	FcεRI	35/47	0.31 (0.01–4.36)
	CD23	1/47	2.65
	IgE on FcεRI+	28/47	0.13 (0.01–1.86)
	IgE on CD23+	1/47	0.17

Number of positive cells, median and range of % positive cells among the respective population is shown. If n = 1, the % positive cells in this patient is displayed.

**Figure 2:**
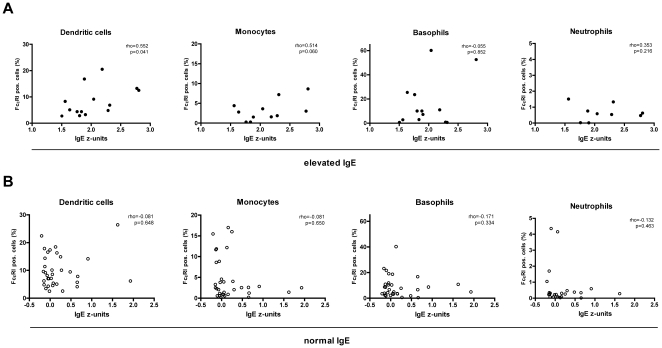
Serum IgE enhances FcεRI on dendritic cells and monocytes from patients with elevated IgE. **A**. Cell surface levels of FcεRI on dendritic cells and monocytes correlate with serum IgE in children with elevated IgE. **B**. No correlation was found in patients with normal serum IgE. Data points represent percent (%) positive cells among cell type after subtraction of isotype control background in individual patients.

Patients with elevated serum IgE (n = 14) showed a significant correlation between age-normalized serum IgE z-units and FcεRI on dendritic cells (rho = 0.552, p = 0.041, Figure 2A). A tendency towards correlation between expression and z-units on monocytes was observed (rho = 0.514, p = 0.06, Figure 2A). FcεRI on basophils and neutrophils showed no association with serum IgE (Figure 2A), nor did FcεRI expression levels in children with normal IgE (n = 34, Figure 2B).

Numbers of CD23+ cells were lower than FcεRI+ cells (Table 2). DCs from 20 and monocytes from 11 out of 48 patients expressed this receptor (0.03%–4.25% CD23+ DCs and 0.29–6.96% CD23+ monocytes in individual subjects). One patient expressed CD23 on basophils and another one on neutrophils. Surface CD23-levels did not correlate with serum IgE (IU/l or z-units) on any of the cell types analyzed in this study (data not shown).

### Cell surface-bound IgE correlates with serum IgE and IgE-receptor expression

FcεRI+ DCs from 45 of 48, FcεRI+ monocytes and basophils from 41 of 48, as well as FcεRI+ neutrophils from 28 of 47 patients stained positive for IgE (Table 2). Percentages of cell surface-IgE positive cells ranged from 0.02 to 17.5% in DCs, 0.1 to 9.66% in monocytes, 0.01 to 61.34% in basophils, and 0.13 to 1.86% in neutrophils. In agreement with our analysis on CD23 expression, we found lower numbers of IgE-loaded CD23+ cells (DCs 17/48, 0.02–4.18% IgE-loaded cells; monocytes 12/48, 0.01–3.92% IgE-loaded cells; basophils 1/48, 1.8% IgE-loaded cells, and neutrophils 1/47, 0.17% IgE-loaded cells).

On FcεRI+ DCs, monocytes, and neutrophils, a positive correlation between cell-bound IgE and absolute total serum IgE was detected (IU/l, Table 3A). Accordingly, age-normalized IgE levels in z-units correlated positively with IgE on the surface of FcεRI+ DCs and monocytes (rho = 0.415, p = 0.003 and rho = 0.354, p = 0.013, respectively, Table 3A). Cell-bound IgE on CD23+ cells did not correlate with serum IgE.

**Table 3:** Cell surface-bound IgE correlates with serum IgE and IgE-receptor expression.

A			Cell-bound IgE on FcεRI+ cells				Cell-bound IgE on CD23+ cells		B	Cell-bound IgE			
	IgE (IU/ml)	IgE (z-units)	DC	Monocytes	Basophils	Neutrophils	DC	Monocytes		DC	Monocytes	Basophils	Neutrophils
**IgE (IU/ml)**	1.0	0.747*	0.653*	0.514*	−0.003	0.429*	0.000	−0.097	**FcεRI+ cells**	0.442*	0.477*	0.662*	0.286
**IgE (z-units)**	0.747*	1.0	0.416*	0.349*	0.161	0.246	−0.44	−0.04	**CD23+ cells**	0.500*	0.702*		

**A**. Surface-bound IgE in FcεRI+ DC, monocytes, and neutrophils correlates with serum IgE. **B**. FcεRI-expression correlates with surface-bound IgE in FcεR+ DC, monocytes, and basophils, CD23-expression correlates with surface-bound IgE in CD23+ DC and monocytes (Spearman rank correlation, respectively).

*p<0.05.

Cell-bound IgE was linked to IgE-receptor expression. FcεRI-expression significantly correlated with cell-bound IgE in FcεRI+ DC's, monocytes, and basophils. CD23-expression correlated with cell-surface IgE in CD23+ DC and monocytes (Table 3B).

### IgE-positive peripheral blood cells are detected in the absence of elevated serum IgE levels

**Figure 3:**
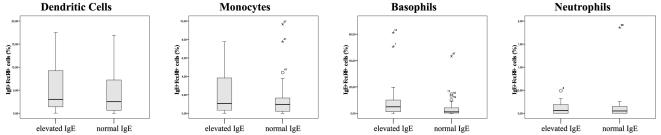
Peripheral blood cells carry IgE, even in the absence of elevated serum IgE. The percentages of IgE-loaded FcεRI+ peripheral blood cells did not significantly differ between patients with elevated versus normal serum IgE (Mann-Whitney-U-test). However, some patients with normal serum IgE showed high levels of cell-bound IgE.

We next analyzed whether elevated serum IgE is reflected by high numbers of cell-bound IgE. We found that numbers of IgE-positive cells did not significantly differ between children with elevated and normal serum IgE (Mann-Whitney-U test, Figure 3). We found, however, a considerable number of patients with high levels of cell-bound IgE on their monocytes, basophils, and neutrophils despite normal serum IgE. These data provide evidence that the body IgE-pool consists of a soluble serum IgE fraction and a circulating cell-bound fraction.

## Discussion

In this work we have correlated levels of cell-bound IgE on peripheral blood cells of pediatric patients with total serum IgE and IgE-receptors. Analyzing expression of the high affinity IgE receptor FcεRI, we observed a slight increase of FcεRI expression on DC and monocytes with age, which is in line with previous findings by Wada et al. [21]. We found that the majority of FcεRI+ dendritic cells, monocytes, basophils and neutrophils carry IgE on their surface. Serum IgE levels correlated positively with levels of cell-bound IgE. Serum IgE positively correlated with FcεRI levels on dendritic cells and to a lesser extent on monocytes in children with elevated IgE. We also found a population of patients that carry high amounts of cell-bound IgE in the absence of elevated levels of serum IgE.

An association between cell surface density of FcεRI and serum IgE levels has been described for adult atopic patients [7], [8], [9], [22], [23]. In mixed-age pediatric populations, comparing IgE values is complicated by age-related normal ranges for IgE and such a relation has not been described yet. In healthy children, the normal range for serum IgE increases with age, hence absolute IgE levels have a different pathophysiological relevance in different age groups. Comparing absolute values would not account for these age-dependent cut-off levels. This problem is commonly solved by converting absolute IgE levels to age-normalized z-values as published by Kjellman et al. [20]. Using age-related z-units, we here demonstrated that high levels of serum IgE are associated with high surface expression of FcεRI in children as well. Another novel finding of this study is the close positive correlation between cell-bound IgE and FcεRI on DC, monocytes, and basophils in children. Our findings support previous data showing that serum IgE is associated with an upregulation of FcεRI as a consequence of IgE binding and stabilization of the receptor complex on the cell surface [4], [5], [6]. In line with previous reports, we showed that FcεRI on neutrophils in our study population was neither regulated by serum- nor cell-bound IgE. This adds to the notion that IgE does not affect FcεRI expression on neutrophils [24], [25].

CD23 expression was not associated with serum IgE, which is consistent with a previous report [10]. Hence, serum IgE does not seem to be involved in regulation of CD23 expression. CD23 expression was, however, correlated with cell-bound IgE. Receptor stabilization by IgE has not been described for CD23 yet. It has however been described that IgE-bound CD23 is shedded from the cell surface and that a soluble form of CD23 is found in patients with high levels of serum IgE [1]. As flow cytometry only allows for the identification of IgE and IgE–receptor double-positive cells, we cannot assess if a receptor is in fact loaded with IgE. We thus cannot rule out that CD23+ cells actually bind IgE via FcεRI. To our knowledge, it has also not been studied whether FcεRI and CD23 expression are interrelated. One could speculate that IgE-mediated surface stabilization of FcεRI synergistically regulates CD23 expression to maximize sensitivity towards IgE and IgE-allergen complexes.

In our study, cell-bound IgE on FcεRI+ cells generally correlated well with elevated serum IgE. In a subgroup of patients though, monocytes, basophils, and neutrophils were loaded with IgE despite normal or low serum IgE levels. Allergic diseases in the absence of elevated serum IgE levels have been described in the literature [18], [19]. We conclude from our data that free serum IgE does not necessarily reflect the body IgE pool. In support of this idea, Liang et al. recently showed IgE-positive cells in peripheral blood. As in our pediatric population, Liang et al. found in healthy and allergic adults that, cell surface-bound IgE and serum IgE correlate well. In line with our findings, this study also observed a discordance in serum IgE and cell-bound IgE in some individuals [26]. We thus propose that the body IgE-pool is composed of a soluble serum IgE fraction and a circulating cell-bound fraction. IgE bound to the high affinity IgE-receptor, FcεRI, on immune effector cells can travel to sites of allergen contact like mucous membranes and cause allergic symptoms. In patients with normal serum IgE as defined by conventional IgE-assays, cell surface bound IgE could sensitize immune cells and trigger allergic reactions. Unfortunately, no data on physician-diagnosed allergies was available for our study population. Due to this limitation of our study, we can only speculate about the role of cell-bound IgE in allergic conditions at this point. Further prospective studies are currently being performed to address this important topic.

Comparable to the increase in FcεRI expression by elevated serum IgE, monoclonal anti-IgE antibodies, such as Omalizumab, decrease serum IgE and FcεRI-surface expression on dendritic cells, monocytes, and basophils [27], [28], [29], [30]. Lower levels of surface FcεRI consequently render immune cells less sensitive to allergen stimuli [31]. Determining cell-bound IgE could be a valuable additional parameter in monitoring IgE-sensitized immune cells during anti-IgE therapy. Overall, measuring cell-bound IgE could be particularly useful in estimating the allergy risk of children with normal serum IgE levels.

In summary, we find that in pediatric patients, levels of age-adjusted serum IgE and IgE-receptors correlate. The majority of FcεRI+ cells also carry IgE on their surface. Cell-bound IgE on FcεRI+ cells correlated well with high levels of serum IgE. We also identified pediatric patients who carried IgE at the surface of peripheral blood cells while serum IgE levels were normal. Further prospective cohort studies are needed for a detailed analysis of the correlation between cell-bound IgE with clinical symptoms of allergy and therapeutic responses. We propose here that measuring cell-bound IgE could be a valuable additional laboratory parameter in the assessment of IgE-mediated allergies.
